# Isolation and pathogenicity of a novel recombinant pseudorabies virus from the attenuated vaccine and classical strains

**DOI:** 10.3389/fvets.2025.1579148

**Published:** 2025-03-17

**Authors:** Zhendong Zhang, Qingteng Wei, Chengyue Wu, Zhengqin Ye, Liting Qin, Ting Chen, Zhe Sun, Kegong Tian, Xiangdong Li

**Affiliations:** ^1^Jiangsu Co-innovation Center for Prevention and Control of Important Animal Infectious Diseases and Zoonoses, College of Veterinary Medicine, Yangzhou University, Yangzhou, China; ^2^College of Veterinary Medicine, Nanjing Agricultural University, Nanjing, China; ^3^Qingdao Jiazhi Biotechnology Co. Ltd., Qingdao, China; ^4^National Research Center for Veterinary Medicine, Luoyang, China

**Keywords:** pseudorabies virus, classical and vaccine strain, evolution, recombination, pathogenicity

## Abstract

Pseudorabies (PR) remains one of the most important swine diseases in China. Live attenuated vaccines have been widely deployed and have proven highly effective in controlling PR in the field. However, recent concerns regarding the evolution and recombination events involving pseudorabies virus (PRV) vaccine strains have raised substantial attention. In the present study, a novel recombinant PRV strain named HN2201 was isolated from one stillbirth case in Henan province in 2022. To assess the genetic and evolutionary features, the major immunogenic and virulence-associated genes, including gB, gC, gD, gG, gE and TK, were sequenced and analyzed. Phylogenetic and nucleotide homology analysis revealed that gB, gC, gD and gG genes of HN2201 displayed close relationship with Chinese classical strains. However, the TK gene of HN2201 contained a continuous deletion of 205 nucleotides, sharing the highest nucleotide homology (99.9%) with HB-98 vaccine strain. Additionally, a similar deletion was observed in the promoter region of the gE gene in both HN2201 and HB-98. Pathogenicity studies on 9-week-old piglets demonstrated that HN2201 exhibited attenuated virulence, characterized by transient clinical signs. The above results suggest that the naturally isolated HN2201 likely resulted from recombination events between the PRV classical strain and the HB-98 vaccine strain. Our findings provide valuable insights into the evolution of PRV in China and underscore the necessity of scientific and cautious use of PRV vaccines in the field.

## 1 Introduction

As one of the economically significant diseases in the swine industry, porcine pseudorabies (PR), also known as Aujeszky’s disease, is a highly notifiable infectious disease with the manifestations characterized by reproductive problem in sows, neurological disorders in fetal or suckling piglets and respiratory distress in growing and fattening pigs (1, 2). Although PR has been eradicated in several foreign countries, including the United States, Denmark, Germany, it continues to affect pig industry all over the world and is classified as a second-class animal epidemic in China (3, 4). Pseudorabies virus (PRV), the causative agent of PR, belongs to the family *Orthoherpesviridae*, subfamily *Alphaherpesvirinae*, and genus Var*icellovirus*.^1^ Wild boar and domestic pig are the primary natural hosts and reservoirs of PRV (1–4). Besides, PRV can infect a wide range of mammals and more than 20 human cases have been also confirmed since 2017, posing a severe threat to the public health (5–7).

The double strand linear DNA genome of PRV is about 145 kb, encoding over 70 proteins through approximately 70 open reading frames (ORFs) (8). Among the proteins, the glycoprotein B (gB) is highly conserved and essential for virus entry and transmission across cells, gC plays very important role in the process of virus adsorption and gD can bind receptors to help the virus to penetrate cells and achieve infection (7). Additionally, the gE and thymidine kinase (TK) proteins play a pivotal role for the virulence of PRV, consequently, they are often targeted to be deleted for genetically developing attenuated live vaccines, with the example of gE-deficient Bartha-K61vaccine and first engineered TK-deleted Bucharest strain (9). Based on the immunogenic and virulence-associated features, gB, gC, gD, gE, and TK are commonly used as markers for studying the evolution and characterization of PRV isolates. Before 2011, PR was effectively controlled in China by the widespread use of Bartha-K61vaccine, which was imported from Hungary in 1979. However, the PR outbreaks have been reported frequently and spread rapidly due to the emergence of PRV variants in late 2011, which were characterized by the amino acid deletion and insertion in gB, gC, gD, gE, and other proteins (10–12). Currently, based on the gC gene, PRV strains are classified into genotype I (commonly foreign strains, such as Bartha and Becker) and genotype II (predominant early SC, Ea, Fa and present HeN1, ZJ01, JS-2012 strains in China) (13, 14). Many studies have shown that Bartha-K61 vaccine (genotype I) could not provide sufficient protection against the newly emergent PRV variants (genotype II). Consequently, several artificially live modified vaccines, SA215 (gE/gI/TK deletion) based on the Fa strain, HB-98 (gE/gG/TK deletion) based on the Ea strain, C (gI/gE/Us9/Us2 deletion) based on C strain and TP (TK/gI/gE/Us9/Us2 deletion) based on HeN1 strain were licensed (15–17).

There is no doubt that the application of PRV vaccines has greatly contributed to the control of PR in China, but the natural recombination events have been reported between modified live vaccine virus and field strains. For example, Ye et al. (18) found the early SC strain may have originated from recombination events between genotype I and early genotype II strains, and Bo et al. (19) provided solid evidence that JSY13 is a natural recombinant of Bartha-K61 vaccine and new variant strain. In 2022, HN2019 strain was isolated and identified as a naturally recombinant PRV between classical strain and the HB-98 vaccine strain (20). In this study, we isolated and characterized a novel recombinant PRV strain, designed HN2201, which appears to have emerged from recombination between classical and vaccine strains. The pathogenicity of HN2201 on growing pigs was evaluated, shedding light on the potential risks posed by natural recombination events involving PRV.

## 2 Materials and methods

In January 2022, a case of abortion in late pregnancy occurred in HB-98 (one commercial PRV vaccine used in China) vaccinated pig farms in Henan Province, China. The abortion rate in the affected batch was approximately 12%, with nearly 100% stillborn piglets. Lung, spleen, brain, lymph node and tonsil tissues of stillborn piglets were acquired and pooled for pathogen screening. Only PRV was identified to be positive, with no other common pathogens, such as porcine reproductive and respiratory syndrome virus, porcine circovirus 2, etc. Then the homogenate of mixed tissues was filtered through 0.22 μm filter and inoculated into Vero cells cultured in 6-well plates for virus isolation, which were cultured in Dulbecco’s modified Eagle medium (DMEM) (Gibco, Shanghai, China) supplemented with 2% fetal bovine serum (FBS, NULEN BIOTECH, Shanghai, China) at 37°C in a humidified incubator under 5% CO_2_. The cytopathic effects (CPEs) were monitored every 6 h, and the infected cells were harvested when CPEs were observed approximately 80%. PRV specific EP0 antibody, generously gifted by Dr. Dongjie Chen (Chinese Academy of Inspection and Quarantine, Beijing), was used to confirm the isolated virus through the indirect immunofluorescent assay (IFA) as previously described (21). The virus was purified using the limited dilution method twice, which then was propagated for viral titer determination calculated as 50% tissue culture infectious dose per mL (TCID_50_/mL) using the Reed-Muench method. To further investigate the growth characteristics of the isolated strain, the monolayer of Vero cells was infected with the purified virus at a multiplicity of infection (MOI) of 0.01 for 1 h. After washing, the fresh DMEM containing 2% FBS was added, and the supernatant and cells were harvested every 12 h post infection for titer determination.

The genomic DNA of PRV was extracted according to the manufacturer’s instruction of Virus Genomics DNA Kit (Tiangen, Beijing, China). The specific primers were designed and used to amplify six major virulence-related genes: gB, gC, gD, gE, gG and TK. The purified PCR products were subjected to Shenggong (Shanghai, China) for sequencing with Sanger method and the acquired sequences were submitted to GenBank database. The corresponding gene sequences from 13 representative PRV reference strains, including genotype I foreign strains, genotype II Chinese classical and variant strains, were download for comparative analysis. Nucleotide and amino acid alignment were performed using the MegAlign model in DNASTAR Lasergene 7. Phylogenetic trees were constructed using MEGA 7.0 with the maximum likelihood (ML) method, the Kimura 2-parameter model and 1,000 bootstrap replication. The referenced strains are listed in Table 1 and the primers used are provided in Table 2.

**Table 1:** Information of PRV reference strains in this study.

Strain	GenBank accession	Country	Isolate year	Genotype (I/II)	Species
Bartha	JF797217	Hungary	1961	I	Pig
Becker	JF797219	USA	1970	I	Pig
SC	KT809429	China	1986	II	Pig
Ea	KU315430	China	1990	II	Pig
Fa	KM189913	China	2012	II	Pig
HB1201	KU057086	China	2012	II	Pig
HeN1	KP098534	China	2012	II	Pig
HN1201	KP722022	China	2012	II	Pig
JS-2012	KP257591	China	2012	II	Pig
TJ	KJ789182	China	2012	II	Pig
ZJ01	KM061380	China	2012	II	Pig
HLJ8	KT824771	China	2013	II	Pig
hSD-1/2019	MT468550	China	2019	II	Human
HB-98-TK	AF469487	China	2002	II	Synthetic^*^

^*^, deletion of portion of TK gene to construct TK−/gG-/LacZ+ pseudorabies virus Ea mutant.

**Table 2:** Primers used for PCR amplification in this study.

Primer	Sequence 5′-3’	Length
gB1-F	ATGCCCGCTGGTGGCGGTC	1,611
gB1-R	GAACTGCAGGCGCGCAAACT	
gB2-F	GCTGATCTCGAACGAGC	1,325
gB2-R	TCTCGAGGCGCTGGTAGT	
gC-F	ATGGCCTCGCTCGCGCGTGCGATGCTCG	1,464
gC-R	TCACAGCGCGGACCGGCGGTAGTAGACGCACGTCG	
gD-F	ATGCTGCTCGCAGCGCTATTGGCGGCGCTGGTCG	1,209
gD-R	CTACGGACCGGGCTGCGCTTTTAGCTCGTCG	
gE-F	ATGCGGCCCTTTCTGCTGCG	1,500
gE-R	GAACTCGTCCTCGGSGTCCA	
gG-F	ATGAGATACTCAACTTTGGAATGT	1,695
gG-R	TAAGCAAGGCCGTACGCAAA	
TK-F	TCGTAGAAGCGGTTGTGGC	1,134
TK-R	GGCAAACTTTATTGGGATGA	

The animal experiment was approved by the animal welfare and ethics committee of Yangzhou University (202409007). All animals were handled following the Experimental Animal Regulation Ordinances defined by the Chinese National Science and Technology Commission. Humane care and healthful conditions were provided for the animals.

Eight 9-week-old healthy piglets, free of PRV antigen and antibody, were selected and randomly divided into two groups in separate rooms throughout the study. The pigs in challenge group (*n* = 4) were each intranasally inoculated with 2 mL HN2201 (10^6^ TCID_50_/mL), while the control group (*n* = 4) was inoculated intranasally with 2 mL of DMEM. Following inoculation, the pigs were monitored twice a day. Rectal temperature and clinical signs were recorded daily for 8 days as previously described (22). The average daily weight gain (ADWG) was calculated by 7 days post inoculation (DPI). Pigs that succumbed during the experimental period, as well as all surviving pigs by 8 DPI were humanely euthanized and subjected to necropsy. Pathological lesions were assessed, and lung, brain and lymph node tissues with severe lesions were inflated with 10% buffered neutral formalin for pathological and immunohistochemistry examination as described previously (21).

## 3 Results

### 3.1 Isolation and growth characteristics of HN2201

**Figure 1:**
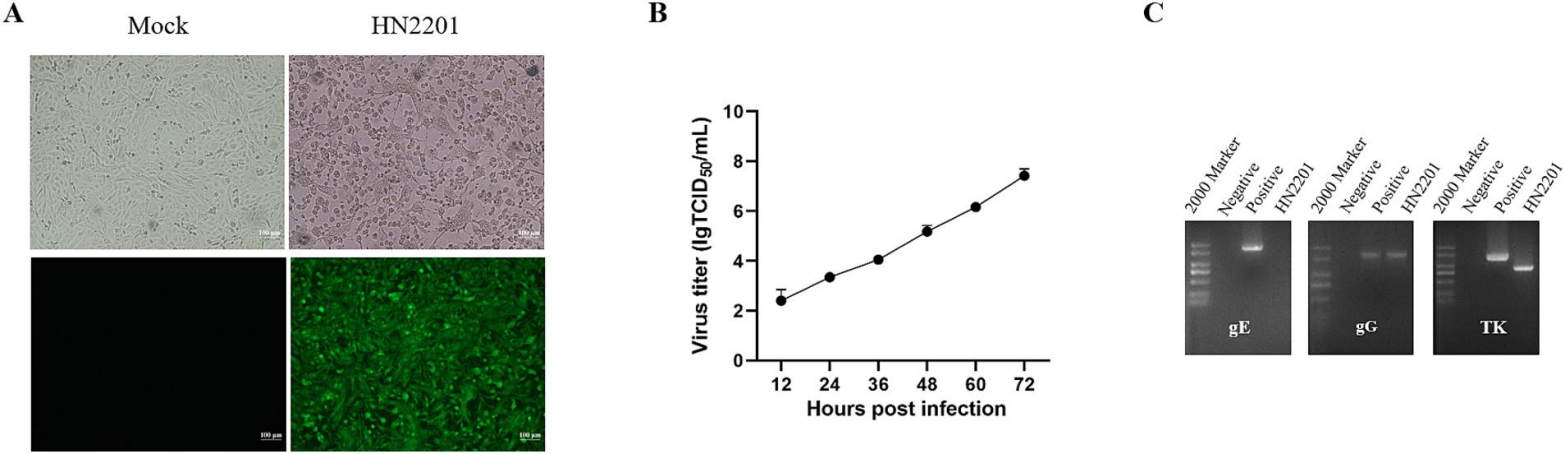
Isolation and identification of PRV HN2201 strain. **(A)** The cytopathic effects (CPEs) and indirect immunofluorescent results (IFA) of HN2201 in Vero cells at 48 h post infection. **(B)** Growth curve of HN2201 in Vero cells at an MOI of 0.01. **(C)** Detection of the PRV gE, gG, and TK genes by PCR, the ddH_2_O and PRV strain JS-2020 (OR271601) were used as negative and positive control, respectively.

Twenty-four hours after inoculation with the supernatants of homogenized PRV positive tissues, the infected Vero cells began to exhibit signs of CPEs, including cell rounding and detachment. By 48 h post infection, typical CPEs were observed in the form of cell fragmentation, rounded and aggregated clumps, but the uninfected cells appeared to be normal (Figure 1A). The presence of infectious PRV was further confirmed via IFA using specific EP0 polyclonal antibody, which showed a strong green fluorescence signal in infected cells (Figure 1A). Following two-round limited dilution, the purified PRV was obtained and designed as HN2201. To better understand the growth features of HN2201 in Vero cells, the growth curve was determined at an MOI of 0.01, and the peak viral titer reached 10^7.5^ TCID_50_/mL (Figure 1B).

### 3.2 Genetic and phylogenetic analysis of major virulent and immunogenic genes of HN2201

**Figure 2:**
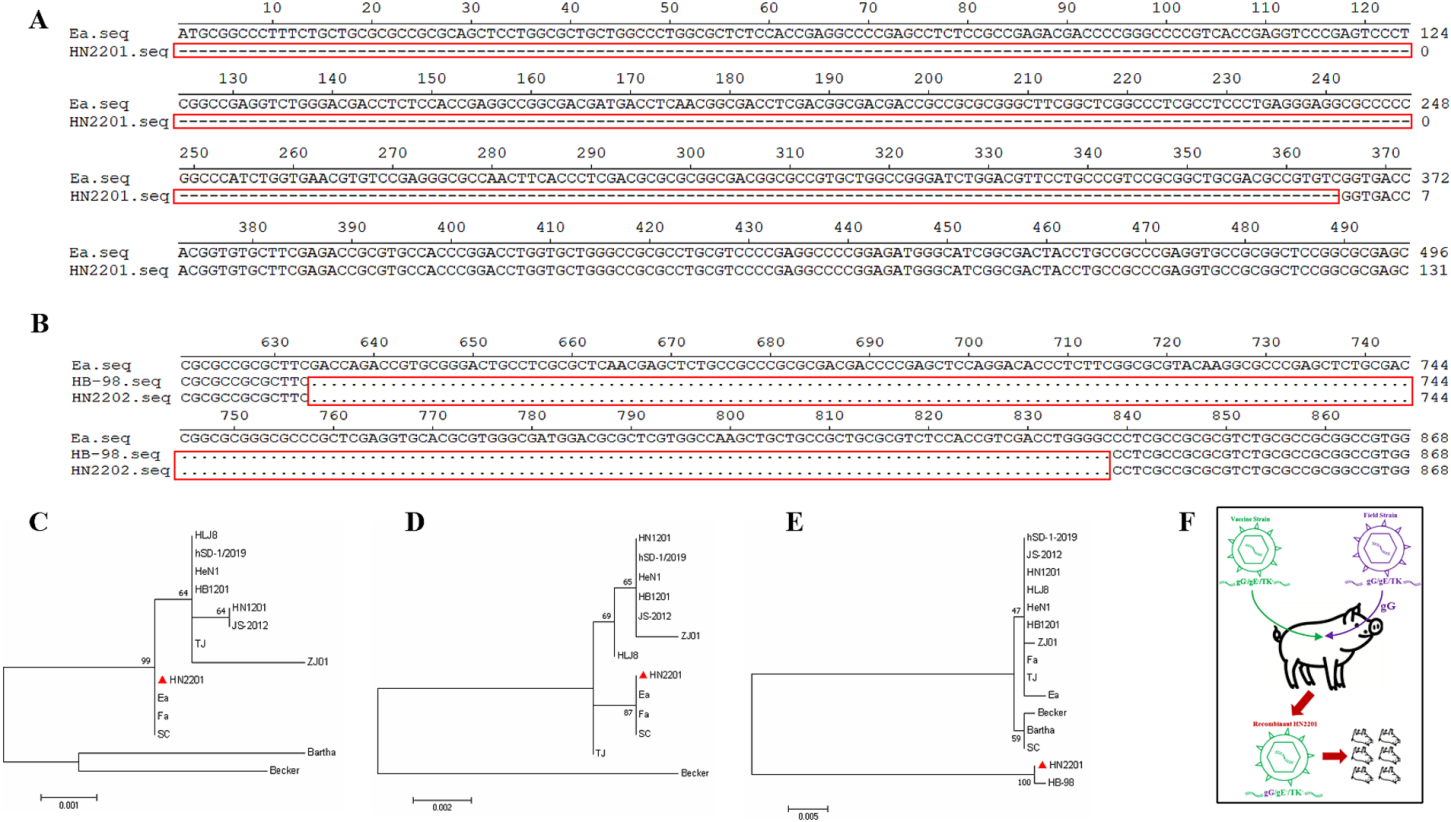
Nucleotide sequence and phylogenetic analysis of gE, gG, and TK genes. Nucleotide sequence alignment of the gE **(A)** and TK **(B)** genes of HN2201, Ea and HB-98 strain. The nucleotide deletions were marked with the red box. **(C–E)** Phylogenetic trees based on the gG **(C)**, partial gE **(D)** and TK **(E)** nucleotide sequences were generated using MEGA7 software with the ML method. **(F)** Proposed model of genomic recombination of HN2201 strain. HB-98-vaccinated pigs were infected with classical PRV strain. HN2201 was produced through acquiring the gG gene during the replication of different PRV strains.

The complete sequences of gE, gG and TK of HN2201 were amplified and analyzed. As shown in Figure 1C, the expected PCR product for the gG gene was acquired, while no amplification was observed for the gE gene used the primers specific to its complete sequences, but a partial sequence lacking ‘promoter region’ was acquired using modified primers (data not shown). For TK, a smaller PCR product was observed than the expected electrophoretic band (Figure 1C). After sequencing, the full length gG, TK and partial-length gE gene sequences were deposited in GenBank with the accession numbers OR161215, OR161216 and OR161214. Nucleotide homology analysis showed that the gG gene of HN2201 shared 100% identity with classical PRV strain (including Ea and Fa), 99.9% identity with variant strain (including HB1201, JS-2012 and HeN1) and 99.2% identity with foreign PRV strain (Bartha). Interestingly, similar to the PCR results, sequence alignment showed continuous deletions of 365 and 205 nucleotides in the gE and TK genes, respectively (Figures 2A,B). These deletions are characteristics of the HB-98 vaccine strain, derived from the Chinese early Ea strain, suggesting that HN2201 may have evolved from the HB-98 vaccine virus. Phylogenetic trees of the gG, TK and partial-length gE genes based on the ML method showed that gG and gE (partial sequence) of HN2201 was clustered into genotype II PRV classical strains, whereas the TK of HN2201 and HB-98 vaccine strain were classified into the same branch (Figures 2C–E). These findings, combining the vaccinated history of HB-98 (gE/gG/TK deletion) derived from Ea strain, supported the hypothesis that HN2201 might be a natural recombinant virus from the classical PRV strain and HB-98 vaccine strain (Figure 2F).

**Figure 3:**
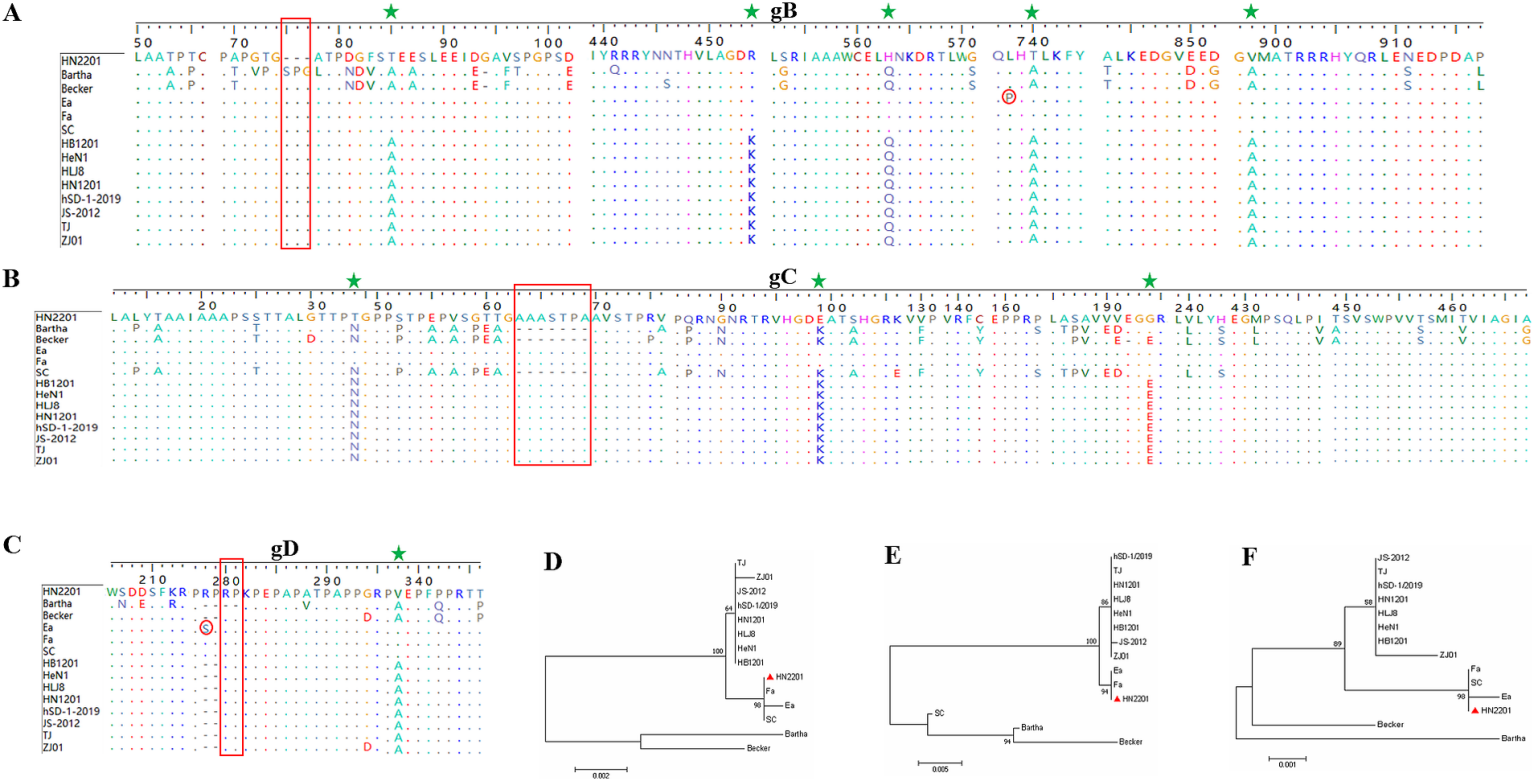
Amino acid and phylogenetic analysis of gB, gC, and gD genes. Amino acid alignment of the gB **(A)**, gC **(B)** and gD **(C)** genes of HN2201 and referenced strains. The characteristic amino acid deletions of Chinese genotype II PRV strains were marked with the red box comparing to the Bartha strain. The characteristic amino acids specific to the earlier Chinese classical strains (Ea and Fa) were marked with green star. The amino acid mutations of HN2201 were marked with red circle comparing to the Ea strain. **(D–F)** Phylogenetic trees based on the gB **(D)**, partial gC **(E)** and gD **(F)** nucleotide sequences were generated using MEGA 7.0 software with the ML method.

Moreover, the gB, gC and gD genes of HN2201 were amplified and sequenced with the accession numbers OR161211, OR161212 and OR161213. Comparative genetic and amino acid analysis of the three major immunogenic genes revealed that HN2201 gB, gC and gD had the highest nucleotide and amino acid homology with classical strain, and lowest homology with foreign genotype I strain (Bartha) (Table 3). Compared to the Bartha strain, HN2201 displayed characteristic amino acid deletions (^75^SPG^77^) in gB, an insertion (^63^AAASTPA^69^) in gC, and an insertion (^280^RP^281^) in gD, which are identical to Chinese genotype II PRV strains. These results indicate that HN2201 is a representative of the Chinese dominant genotype II strain. Furthermore, five, three and one amino acids variations were identified in gB (^85^T, ^454^R, ^563^H, ^740^T, ^898^V), gC (^34^T, ^99^E, ^194^G) and gD (^297^V), respectively, all of which were specific to the earlier Chinese classical strains (Ea and Fa). These above results further provided strong evidence that HN2201 might be evolved from vaccine virus HB-98. Two amino acid mutation were found in gB (P^738^L) and gD (S^278^R), demonstrating that the vaccine virus has undergone continuous evolution. Phylogenetic trees based on the gB, gC and gD nucleotides showed they were in the same clade with the classical strain, further confirming the evolutionary results of HN2201 that the isolate was derived from HB-98 vaccine virus and underwent recombination event (at least gG gene) with field strain (Figures 3A–F).

**Table 3:** The nucleotide and amino acid identities of gB, gC, gD, gG and TK.

Query gene	Nucleotide/Amino acid identities (%)					
	Classical strain		Variant strain			Genotype I
	Ea	Fa	HB1201	JS-2012	HeN1	Bartha
HN2201-gB	99.7/99.6	99.7/99.8	99.6/99.2	99.6/99.2	99.6/99.2	98/96.4
HN2201-gC	100/100	100/100	99.7/99.4	99.7/99.2	99.7/99.4	95.9/92.9
HN2201-gD	99.8/99.5	100/100	99.6/99.8	99.6/99.8	99.6/99.8	98.7/97.3
HN2201-gG	100/100	100/100	99.9/99.8	99.9/99.8	99.9/99.8	99.2/99.2
HN2201-TK	93.1/89.3	93.3/89.3	93.3/89.3	93.3/89.3	93.3/89.3	93.3/89.3

### 3.3 Pathogenicity study of HN2201 on 9-week-old pigs

**Figure 4:**
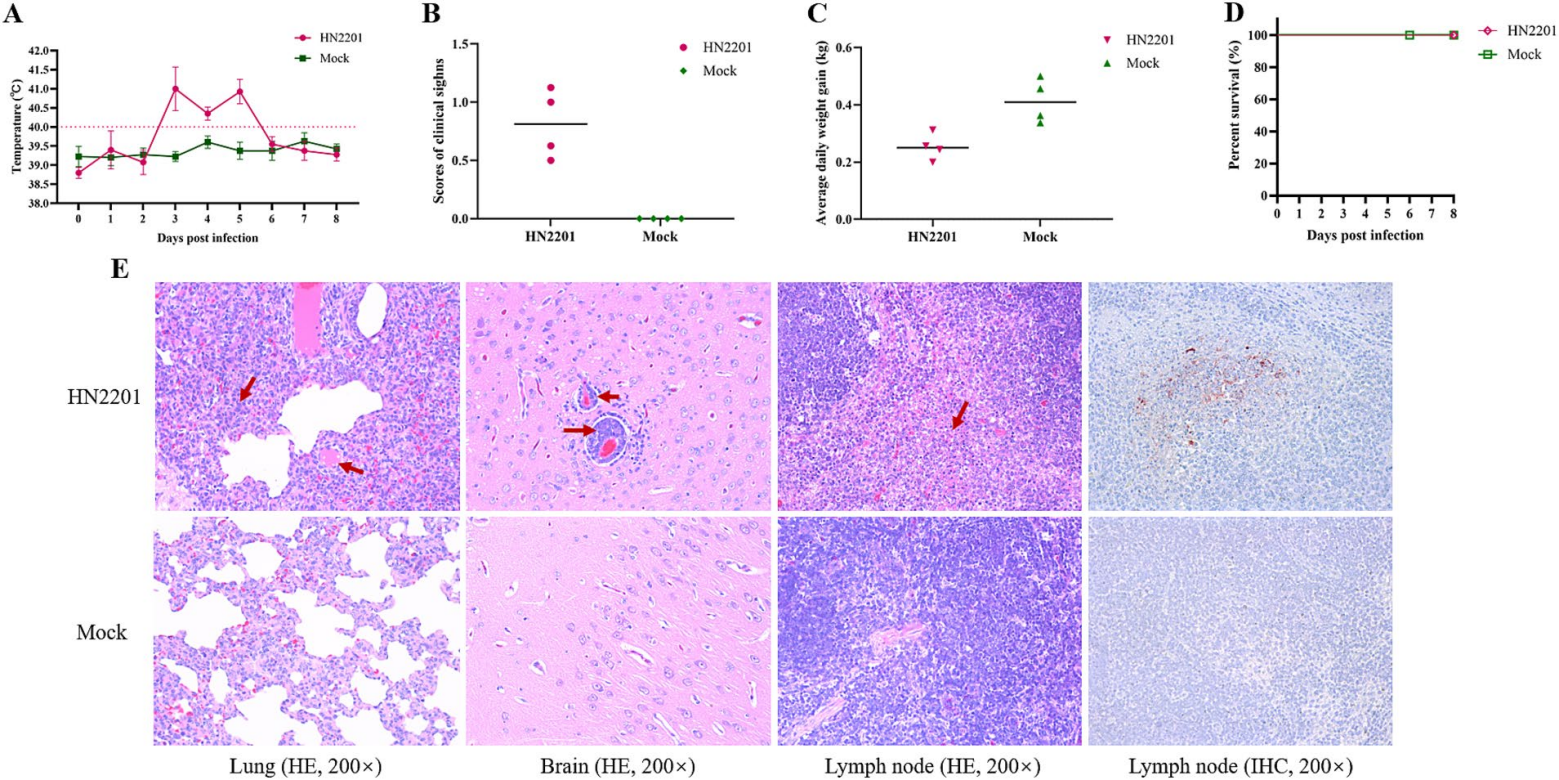
Pathogenicity of HN2201 in piglets. Nine-week-old health piglets were infected with 2 mL of HN2201 (10^6^ TCID_50_/mL). The daily temperature **(A)**, scores of clinical signs **(B)**, average daily weight gain **(C)**, and survival percent **(D)** were recorded and analyzed, respectively. **(E)** Histopathological examination (HE) and immunohistochemistry staining (IHC) results of HN2201 infected or mock piglet. Original magnification 200 × .

To assess the pathogenicity of HN2201, 9-week-old healthy piglets were inoculated with 2 mL of HN2201 (10^6^ TCID_50_/mL) intranasally or DMEM as placebo, and the clinical symptoms of each group was observed daily for 8 days. As shown in Figure 4A, the infected piglets showed high fever (above 40°C) by 3 DPI, persisting for 3 days and recovered by 6 DPI, which accompanied with transient cough, sneeze and respiratory distress (Figure 4B). During the experiment, no piglets died in HN2201 inoculated group but the average daily weight gain was significantly lower compared to mock group (Figures 4C,D). Additionally, the microscopic histopathological examination and immunohistochemistry staining were performed to further evaluate the virulence of HN2201 after autopsy by 8 DPI. As shown in Figure 4E, the interstitial pneumonia characterized by alveolar interstitial thickening and inflammatory cell infiltration, as well as nonsuppurative encephalitis marked with the phenomenon of vascular neural sheath (inflammatory cells infiltration around the small blood vessels) and formation of large necrotic foci in lymphoid tissue were observed in HN2201 challenged group, but there were no obvious microscopic lesions in the mock group. Immunohistochemistry staining revealed that PRV antigens were positive in lymphoid tissue of infected pigs (Figure 4E).

## 4 Discussion

PRV is one of the most critical pathogens threatening the global pig industry. As the world’s largest pig producer and consumer, China has been combating the PRV pandemic since the 1970s and experienced severe and large scale outbreaks of PR occurring at the end of 2011, coinciding with the emergence of variant PRV, which marked the new milestone in the evolution of PRV in China (23). Of particular concern, hSD-1/2019 was successfully isolated firstly from the patient with acute encephalitis, and many human PRV cases have been reported in recent years (7, 24). So, it is of great importance to continuously monitor and understand the current status and evolution of PRV strains in China.

Vaccines have been a cornerstone strategy in controlling PR in the field. The first live modified PRV vaccine named Bartha-K61, which carries a gE/gI deletion, not only provided very efficacious protection but enabled the eradication of PRV due to the DIVA (Differentiation Infected from Vaccinated Animals) concept (25). Between the 1990s and late 2011, effective PR control was achieved by vaccination with Bartha-K61 in the most of Chinese pig farms (2). Nevertheless, the emerged variant PRV strain in 2011 circumvented the vaccine protection and spread rapidly across China, resulting in huge economic losses. In response to the new challenge, the licensed SA215 (derived from Fa strain with gE/gI/TK deletion), HB-98 (derived from Ea strain with gE/gG/TK deletion) and modified Bartha-K61 with enhanced quality, along with improved biosecurity measures and vaccination schedules, were applied to control the PR in China. Fortunately, the PRV gE antibody and sequence positivity rate gradually decreased from 39.92 and 14.75% at peak to 15.38 and 1.52% in 2021, respectively (23). Despite the success of vaccination programs, live modified PRV vaccines are not without limitations (26, 27). It was speculated that the variation in the major immunogenic and virulent proteins, as well as the emergence of the variant strain, are due to the long-term immune pressure exerted by the widespread use of Bartha-K61 since the 1990s (28). Moreover, recombination events between field strains and vaccine strains have been increasingly recognized as a contributing factor to PRV evolution.

Recombination is a key mechanism driving virus evolution, such as African swine fever virus, porcine reproductive and respiratory syndrome virus, and porcine epidemic diarrhea virus (29–31). There was a high recombination rate *in vivo* when different PRV strains were co-infected sheep and pigs (32). Wang et al. analyzed 55 PRV genomes, 23 recombination events were identified and 16 events were observed from Bartha-K61 and Chinese strains (28). Similarly, it has been reported that the SC, JSY13 were recombinants from Bartha-K61 and Chinese early local and variant isolate, respectively (18, 19). Furthermore, He et al. (33) found that HeN1 and Qihe547 were originated from recombination events between vaccine virus derived from genotype II Chinese classical strain (Ea and Fa) and genotype I foreign strain, and HN-2019 was also identified a naturally recombinant from the field virus and HB-98 vaccine strain (20). In the present study, a novel field PRV strain designed HN2201 was successfully isolated in Henan province, China. The full length sequence of major immunogenic and virulent genes, including gB, gC, gD, gE, gG and TK, were determined. According to the phylogenetic trees of the six genes and reference strains, we found the virus was an earlier PRV. The genetic analysis of gG, gE and TK genes revealed that gG had 100% nucleotide homology with classical PRV strains (Ea and Fa) (Table 3), while the homology of the HN2201 TK gene with HB-98 vaccine strain was the highest 99.9% (only a nucleotide difference at the position of 13), similarly with a continuous deletion of 205 nucleotides (20). Notably, HN2201 and HB-98 shared the same deletion pattern in the gE gene sequence. Together with the fact that HB-98 vaccine characterized by triple gene deletion (gE/gG/TK) was used in the pig farms, therefore, it is logical to believe that HN2201 acquired its genome sequence at least from two origins: HB-98 vaccine strain and the classical field strain. Whether other inter or intra-clade recombination event occurs, high-throughput sequencing technologies are warranted to investigate potential additional recombination events across the HN2201 genome. Besides, in light of the increasing cases of recombinant PRV strains derived from vaccine viruses and the need to mitigate PRV genetic diversity, it is imperative to implement scientific vaccination strategies. Specifically, PRV MLV should be restricted to gE-negative pig farms or discontinued entirely. Excessive and prolonged use of PRV MLV, even in PRV-endemic farms with unstable infection status, should be avoided to minimize the risk of viral recombination and evolution.

In the field, the natural infection of PRV can cause varying clinical signs, with severity dependent on the virus strain and the age of pigs. In general, the clinical symptoms would become lighter along with the increase in pig age. The PRV challenged animal experiments can be conducted in various animals. Tan et al. (20) performed the animal experiments of HN-2019 (also recombinant from the field virus and HB-98 vaccine strain) in mice and exhibited 33.33% mortalities. Due to the costs and inconvenience, little pathogenicity evaluations were conducted in pigs. Yang et al. (34) previously showed that intranasal infection is more effective than intramuscular infection for pig model. Here, we evaluated the virulence of HN2201 in 9-week-old pigs intranasally. Pigs infected with the recombinant HN2201 lacking gE and TK protein showed transient and mild clinical symptoms, along with noticeable microscopic lesions. Given the gE and TK gene are responsible for PRV virulence, gE or TK deleted PRV strain typically showed attenuated pathogenicity compared with the wild strains, which was highly in accordance with our results and further confirmed our speculation that HN2201 derived from vaccine virus. Although no death occurred, a decrease of nearly two fold of average daily weight gain was observed, implying the detrimental effect of HN2201 for weaning to finished pigs. Even worse, under poor management and co-infection or secondary infection of other pathogens in the field, the complexity and production performance would be exacerbated. Besides, since HN2201 was isolated from stillbirths in pregnant sows, the pathogenicity of HN2201 for sows might be more virulent and need to be evaluated in the future.

## 5 Conclusion

In summary, a novel PRV strain recombinant from classical and attenuated vaccine strain was isolated and analyzed in the present study. Our results provide important evidence for PRV recombination events associated with vaccine virus in the field and highlight the significance for continuously monitoring PRV evolution. Moreover, PRV modified live vaccines should be used scientifically on unstable PRV-infected pig farms and eradication programs should be implemented and expanded furtherly in China.

## Data Availability

The datasets presented in this study can be found in online repositories. The names of the repository/repositories and accession number(s) can be found in the article/supplementary material.

## Footnotes

^1^ https://ictv.global/taxonomy
